# Transformation Mechanism of Rare Ginsenosides in American Ginseng by Different Processing Methods and Antitumour Effects

**DOI:** 10.3389/fnut.2022.833859

**Published:** 2022-04-04

**Authors:** Zhi-man Li, Zi-jun Shao, Di Qu, Xiao-hui Huo, Mei Hua, Jian-bo Chen, Yu-shun Lu, Ji-Yue Sha, Shan-shan Li, Yin-shi Sun

**Affiliations:** ^1^Institute of Special Animal and Plant Sciences, Chinese Academy of Agricultural Sciences, Changchun, China; ^2^Institute of Biological and Pharmaceutical Engineering, Jilin Agricultural Science and Technology University, Jilin, China

**Keywords:** *Panax quinquefolium* L., rare ginsenosides, transformation, S180 tumour-bearing mice, processing methods

## Abstract

The mechanism by which ginsenosides from *Panax quinquefolium* L. transform into rare saponins by different processing methods and their antitumour effects have yet to be fully elucidated. Our study aimed to detect the effect of amino acids and processing methods on the conversion of ginsenosides in American ginseng to rare ginsenosides, using 8 monomeric ginsenosides as substrates to discuss the reaction pathway and mechanism. S180 tumour-bearing mice were established to study the antitumour effects of American ginseng total saponins (AGS-Q) or American ginseng total saponins after transformation (AGS-H) synergistic CTX. The results showed that aspartic acid was the best catalyst, and the thermal extraction method had the best effect. Under the optimal conditions, including a reaction temperature of 110°C, an aspartic acid concentration of 5%, a reaction time of 2.5 h and a liquid-solid ratio of 30 mL/g, the highest conversion of Rk_1_ and Rg_5_ was 6.58 ± 0.11 mg/g and 3.74 ± 0.05 mg/g, respectively. In the reaction pathway, the diol group saponins participated in the transformation process, and the triol group saponins basically did not participate in the transformation process. AGS-Q or AGS-H synergistic CTX, or AGS-H synergistic CTX/2 could significantly increase the tumour inhibition rate, spleen index and white blood cell count, had a significant upregulation effect on IL-2 and IL-10 immune cytokines; significantly restored the ratio of CD4^+^/CD8^+^; and significantly inhibited the level of CD4^+^CD25^+^. AGS-Q or AGS-H synergistic with CTX or CTX/2 can significantly upregulate the expression of Bax and cleaved-Caspase-3 and inhibit the expression of antiapoptotic protein Bcl-2. AGS synergistic CTX in the treatment of S180 tumour-bearing mice can improve the efficacy and reduce toxicity.

## Introduction

*Panax quinquefolium* L. is an herbaceous plant of the genus, also called American ginseng, which originates from the United States and Canada (1). Shandong, Jilin, and Heilongjiang in China are now the main areas producing this plant. Ginsenoside is the main active component of American ginseng and can be divided into dammarane type and oleanolic acid type according to structure. Among dammarane ginsenosides, tetracyclic triterpenoids can be further divided into protopanaxadiol (PPD) types, such as Rb_1_, Rb_2_, Rc, Rd, and protoparitol (PPT) types, such as Re and Rg_1_ (2, 3). In addition, there are some ginsenosides in American ginseng that have a low content of raw materials, such as 20(S)-Rg_3_, 20(R)-Rg_3_, Rk_1_ and Rg_5_. Natural ginsenosides have the characteristics of high polarity and large molecular weight, and are not easily absorbed through the intestinal mucosa. Instead, they are converted into rare ginsenosides under the action of enzymes secreted by specific flora in the intestinal tract, which are absorbed and utilized by the body, and then exert medicinal effect. Therefore, that obtains rare ginsenosides with better activity through *in vitro* biotransformation of natural ginsenosides is also one of the directions pursued by researchers in the industry.

Some studies have shown that rare ginsenosides, Rk_1_ and Rg_5_, have significant effects on some diseases, such as depression (4), diabetes (5), and anti-septicaemia (6), and they have an obvious effect on promoting apoptosis of cancer cells such as liver cancer, lung cancer and gastric cancer (7–10). Shenyi capsules are the first to contain rare ginsenoside monomers, and 20(S)-Rg_3_ prescription drugs are used in clinical anticancer therapy. Due to the beneficial effects of rare saponins Rk_1_ and Rg_5_, researchers have paid more attention to Rk_1_ and Rg_5_, and the market demand has also increased. Therefore, it is important to explore an efficient enrichment method. In some reports, the rare ginsenosides, Rk_1_ and Rg_5_ have been shown to be enriched by cooking and drying processes (11–13). These methods are complicated and time-consuming. In addition, rare ginsenosides Rk_1_ and Rg_5_ can also be increased by acid hydrolysis, microbial degradation, and metal ion catalysis (14–16). However, these methods require high specificity and reaction conditions and cause environmental pollution. Therefore, it is very important to screen environmentally friendly, safe, and efficient catalysts. It has been reported that aspartic acid can degrade the total saponins of purified protopanaxadiol in ginseng by steaming to obtain the rare ginsenosides 20(S)-Rg_3_, 20(R)-Rg_3_, Rk_1_ and Rg_5_ (17). Amino acids are biologically active macromolecules used by organisms to construct and repair tissues. Amino acids also provide energy to the body and brain. Therefore, the selection of a suitable amino acid as a catalyst is of great significance for the safe and efficient conversion of rare ginsenosides. Our study for the first time reported the preparation method of tramforming ginsenosides from American ginseng natural ginsenosides into rare saponins Rk_1_ and Rg_5_, aiming to develop a low-polluting, simple and low-cost conversion route, and verify the anticancer mechanism of the AGS-H. This will have potential value for the development of high-efficiency preparations based on rare ginsenosides, and the large-scale industrial production of rare ginsenosides is of great significance.

Cyclophosphamide (CTX) is an effective anticancer alkylating agent that also has a broad spectrum of cytotoxicity to normal cells. Its metabolites, such as phosphamide mustard (PM) and acrolein (Acr), can interact with DNA to induce the formation of DNA admixtures, resulting in oxidative damage to DNA (18). Many studies have shown that Chinese herbal medicines have great potential in reducing the toxicity of chemotherapy. American ginseng, a well-known traditional Chinese herbal medicine with nourishing effects, contains bioactive ingredients such as saponins, polysaccharides and peptides. In this study, based on the pharmacological effects of ginsenosides on immune regulation, antitumour activity, and anti-inflammatory activity, we investigated the effect of American ginseng total saponins (AGS-Q) or American ginseng total saponins after transformation (AGS-H) on CTX mediated immunosuppression and its antitumour mechanism.

## Materials and Methods

### Materials and Reagents

Aspartic acid (Asp), glutamic acid (Glu), arginine (Arg), histidine (His), and lysine (Lys) were purchased from Soledad Bao (Beijing, China). Ginsenosides Re, Rg_1_, Rb_1_, Rb_2_, Rc, Rd, 20(S)-Rg_3_, 20(R)-Rg_3_, Rk_1_ and Rg_5_ were purchased from Shanghai Yuanye Biotechnology Co., Ltd., China, purity ≥98%. Chromatographic grade acetonitrile and methanol were purchased from Fisher (Waltham, MA, USA) and C_18_ Sep-Pak® SPE was purchased from Race Point Technology (Saifen Technology Co., Ltd., Ireland). An Ultrapure Water System (Water Purifier Co., Ltd., Chengdu, China) was used to obtain ultrapure water. The mouse S180 sarcoma cell line was obtained from Shanghai Institute of Biochemistry and Cell Biology, China. RPMI-1640 medium, fetal bovine serum (FBS), benzylpenicillin, and streptomycin were provided by Gibco (Grand Island, NY, USA). CTX was purchased from Shengdi Pharmaceutical Co., Ltd. (Jiangsu, China). Antibodies against T subpopulations, including fluorescein isothiocyanate (FITC)-conjugated rat anti-mouse CD4, allophycocyanin (APC)-conjugated rat anti-mouse CD8, and phycoerythrin (PE)-conjugated rat anti-mouse CD25, were obtained from BioLegend (San Diego, CA, USA). Anti-Caspase-3 (sc7272) antibody was purchased from Santa Cruz Biotechnology Inc. (Santa Cruz, CA, USA). Anti-Bcl-2 (ab182858), anti-Bax (ab32563), anti-GAPDH (ab8245) and horseradish peroxidase (HRP)- conjugated secondary antibodies were purchased from Abcam (Cambridge, MA, USA).

### Source and Treatment of American Ginseng

The dried samples were purchased from Wanliang Ginseng Market in Fusong County, Jilin Province, China, and identified as American ginseng (4 years old) by Professor Li Wei for Jilin Agricultural University. The medicinal material was pulverized into a powder by a pulverizer (Dingli Medical Instrument Co., Ltd., Wenzhou, China), further processed through a 60-mesh stainless steel mesh and then stored in a cool dry place.

### Amino Acid Screening

Five amino acids, including two acidic amino acids, Asp and Glu, and three basic amino acids, Arg, His, and Lys were chosen. The reaction conditions were a concentration of 5% amino acids, reaction time of 1 h, liquid-solid ratio of 20 mL/g, and a temperature of 120 °C. The extract was collected and filtered through a 0.22-μm filter. The analysis was performed using an Acquity Ultra High-Performance Liquid Chromatography (UPLC) (Waters, Manchester, UK). The process was repeated three times for all samples.

### Optimization of Reaction Conditions

First, four factors were investigated: reaction temperature (70–120 °C), concentration of amino acids (1–20%), liquid-solid ratio (5–50 mL/g), and reaction time (0.5–3 h). Second, according to the results of the above single factor experiments, orthogonal experiments were designed to optimize the conversion parameters (factors). The orthogonal experiments consisted of nine independent factors. The order of the experiments was random to ensure that the results were valid in this study. The process was repeated three times for all samples.

### Optimization of Extraction Method

Four extraction methods were examined: reflux extraction (RE), heat extraction (HE), soak extraction (ME), and ultrasonic extraction (USE) for optimization of the extraction method. All extraction methods involved 2 g of American ginseng powder being accurately weighed. RE (19) involved American ginseng powder, 5% amino acid, and 100 mL of 70% ethanol solution left to react for 1.5 h. The supernatant was collected by filtration and the residue was extracted once, combined with the supernatant, and passed through a 0.22 μm filter. UPLC analysis was then repeated three times. HE (16) involved American ginseng powder and 5% amino acid added to 40 mL of distilled water and left to react at 110 °C for 2 h. The supernatant was collected and filtered through a 0.22-μm filter, and UPLC analysis was repeated three times. ME involved American ginseng powder and 5% amino acid extracted three times in 40 mL of distilled water for 6 h. The supernatant was collected and filtered through a 0.22-μm filter, and UPLC analysis was repeated three times. USE (20) involved American ginseng powder and 5% amino acid in triplicate added to 100 mL of 50% ethanol solution and left to react in a 250-W ultrasonic wave (Hubei Dingtai Biochemical Technology Equipment Manufacturing Co., Ltd.) for 1 h. The supernatant was collected and filtered through a 0.22-μm filter, and UPLC analysis was repeated three times.

### Verification of the Transformation Pathway

Eight pure ginsenosides were selected, including two common PPT types: Re and Rg_1_, four common PPD types: Rb_1_, Rb_2_, Rc, Rd, and two rare ginsenosides 20(S)-Rg_3_ and 20(R)-Rg_3_. The reaction conditions of the optimized ginsenosides Rk_1_ and Rg_5_ were used to simulate and verify their transformation pathways.

### UPLC Analysis

#### Chromatographic Conditions

Columns ACQUITY UPLC® BEH C_18_ (2.1 mm × 50 mm, 1.7 μm); mobile phase of water (A)-acetonitrile (B), elution program (0~5.80 min, 87%~78% A; 5.80~18.75 min, 78%~62% A; 18.75~22.05 min, 62%-60% A; 22.05~23.55 min, 60%~55% A; 23.55~24.25 min, 55%~42% A; 24.25~30.00 min, 42%~38% A; 30.00~30.75 min, 38%~20% A; 30.75~37.75 min, 20%~0% A; 37.75~40 min, 0%~87% A); column temperature was 35 °C; flow rate was 0.4 mL/min; injection volume was 3 μL; detection wavelength was 203 nm.

#### Preparation of the Drawing Standard Curves

First, 5 mg of ginsenosides Rk_1_ and Rg_5_ were weighed and dissolved in methanol in a 5 mL volumetric flask. A mixed standard of 0.1, 0.2, 0.4, 0.8, and 1.6 mL into a 5 mL volumetric flask chromatography methanol solution was prepared. The standard curve was drawn with the concentration as the abscissa and the peak area as the ordinate. The regression equation of ginsenoside Rk_1_ was *Y* = 2.63e + 6*X*-2.38e + 4, *R2* = 0.9996, linear range was 0.0208–0.3328 mg/mL; the regression equation of ginsenoside Rg_5_ was *Y* = 9.36e + 6*X*–8.19e + 4, *R*^2^ = 0.9998, and linear range was 0.0206–0.3296 mg/mL.

### Preparation of Total Saponins From American Ginseng

According to the method of amino acid transforming rare saponins in American ginseng, the optimal transformation conditions were selected. The rare saponins in American ginseng were transformed to obtain the transformed extract. Purification of D101 macroporous resin was performed. Ethanol gradient elution was used to obtain the eluted total saponin solution, which was concentrated and freeze-dried to obtain freeze-dried powder of the total saponin of *Panax quinquefolium* before (AGS-Q) and after the transformation (AGS-H).

### Animals Treatment and Experimental Procedure

SPF ICR mice (18–22 g) were provided by Changsheng Biotechnology Co., Ltd. (Liaoning, China) were raised in a 12 h light/dark cycle at 23 ± 1°C, relative humidity of 50 ± 5% environment. Mice had a free access to water and foods during the adaptation period. All experiments were executed strictly according to the Principle of Laboratory Animal Care and the guidelines prescribed by the Animal Research Committee of the Institute of Special Animals and Plants Sciences, Chinese Academy of Agricultural Sciences (Permit No.: ECLA-ISAP-18079). Each mouse was injected with 0.2 mL S180 cell suspension at a concentration of 1 × 10^5^ cells/mL in the right axilla to establish the S180 tumour-bearing mouse model.

After 24 h, these mice were randomly divided into: the model group, CTX (25 mg/kg), CTX + AGS-QL (25 mg/kg + 100 mg/kg), CTX + AGS-QM (25 mg/kg+200 mg/kg), CTX+AGS-QH (25 mg/kg+400 mg/kg), CTX+AGS-HL (25 mg/kg+100 mg/kg), CTX+AGS-HM (25 mg/kg+200 mg/kg), and CTX+AGS-HH (25 mg/kg+400 mg/kg).

After inoculation, the mice were divided into the model group, CTX group (25 mg/kg), AGS-HL and AGS-HH groups (200 and 400 mg/kg), and CTX/2+AGS-HL group (12.5 mg/kg+200 mg/kg), CTX/2+AGS-HH (12.5 mg/kg+400 mg/kg).

Mice were given AGS daily by gavage and intraperitoneal injection of CTX for 14 consecutive days. Mice in the normal group were intragastrically injected with normal saline. After the last administration, the mice were fasted for 12 h. The mice were anesthetized and sacrificed after blood collection. The tumor and spleen were stripped and weighed, fixed with 10% formalin and stored at−80 °C.

### ELISA Analysis

After the tests, blood were isolated immediately. The serum was separated by centrifugation at 3000 rpm for 30 min at 4 °C. Serum and tissue samples were frozen at −80 °C for subsequent analysis. Mouse IL-2 and IL-10 levels in serum were measured by enzyme-linked immunosorbent assay (ELISA) kits of Ebioscience (California, USA).

### Mouse Leukocyte Assay and Splenic T-Lymphocyte Subpopulation Assay

For the splenic T-lymphocyte subpopulation assay, the splenocyte suspension was adjusted to 1 × 10^6^ cells/mL and subjected to flow cytometry to measure the splenocyte lymphocyte subpopulations. The splenocyte surface markers were labeled with fluorescein isothiocyanate (FITC)-conjugated anti-mouse CD4, APC-conjugated anti-mouse CD8, and PE-conjugated anti-mouse CD25. The labeled cells were washed twice, resuspended in staining buffer (BioLegend), and analyzed using a FACSCalibur (BD Medical Technologies, Franklin Lakes, NJ, USA) and CellQuest software. For comparison, the cells stained with isotype-matched antibodies were used to calibrate the FACSCalibur instrument settings.

### Western Blotting

The protein extracted from tumor with lysis buffer. SDS-PAGE (8–12%) gels were used to separate equal amounts of the protein, and then proteins were transferred onto PVDF membranes. The membranes were incubated with blocking solution (5% skim milk) for 1 h and then specific primary antibodies, including Bax, Bcl-2, cleaved-Caspase-3 and GAPDH antibodies, followed by an HRP-conjugated secondary antibody. Finally, the target proteins were visualized using a BeyoECL Plus Kit (Beyotime Biotechnology, Shanghai, China), and analyzed by densitometry with Image-Pro Plus 6.0 (Media Cybernetics Inc., Rockville, MD, USA).

### Statistical Analysis

Data are represented as the mean±SD. All data's were analyzed using one-way ANOVA and Tukey's multiple comparisons test. Statistically significant differences in p values between groups were considered as <0.05. All statistical analyses were performed using GraphPad Prism version 5 (GraphPad Software, San Diego, CA, USA).

## Results

### Effect of Amino Acids on the Conversion Rates

**Figure 1:**
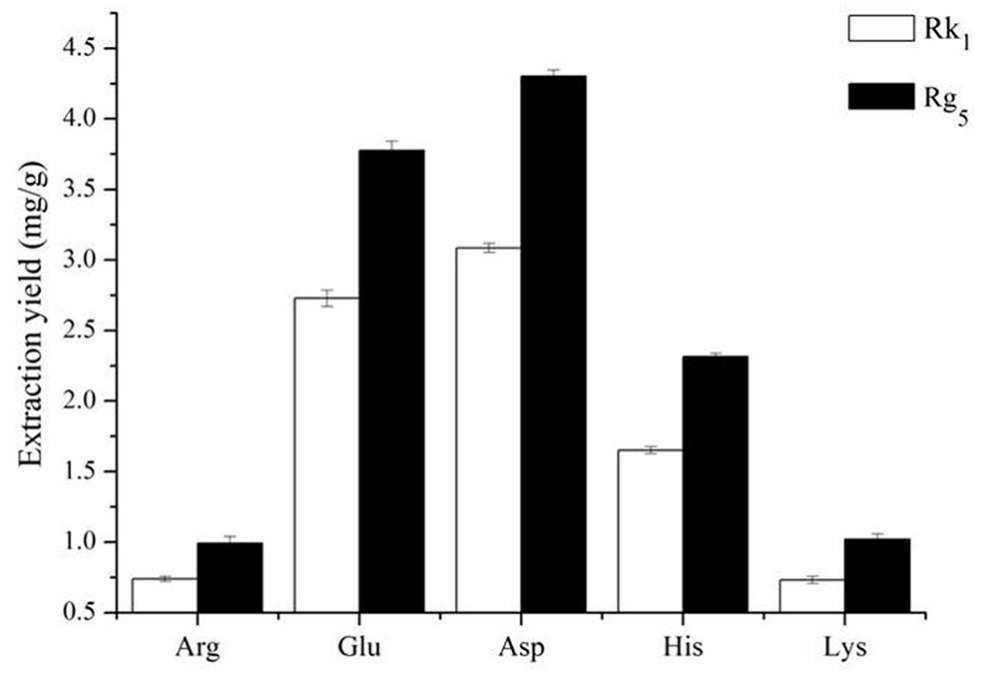
Effect of different amino acids on the contents of ginsenosides Rk_1_ and Rg_5_ (*n* = 3).

The effect of different amino acids on the conversion rates of Rk_1_ and Rg_5_ is shown in Figure 1. The conversion rates of Rk_1_ and Rg_5_ using five different amino acids were significant (*P* < 0.05). The transformation effects of Asp, Glu, His, Lys and Arg on Rk_1_ and Rg_5_ were successively weakened. The transformation of Rk_1_ and Rg_5_ by acidic amino acids was significantly higher than that by alkaline amino acids (*P* < 0.05). The effect of Glu on the conversion rates of Rk_1_ and Rg_5_ was 2.73 ± 0.06 mg/g and 3.78 ± 0.07 mg/g, respectively. The effect of Asp on the conversion rates of Rk_1_ and Rg_5_ was 3.08 ± 0.03 mg/g and 4.30 ± 0.04 mg/g, respectively, with the best conversion rate. This was consistent with results from Xia Juan (21). Compared with strong acids, the conversion rates of rare ginsenosides can be increased by 1.28 times, so Asp was selected as the best catalyst (22).

### Effect of Temperature, Asp Concentration, Liquid to Solid Ratio and Time on the Conversion Rates

**Figure 2:**
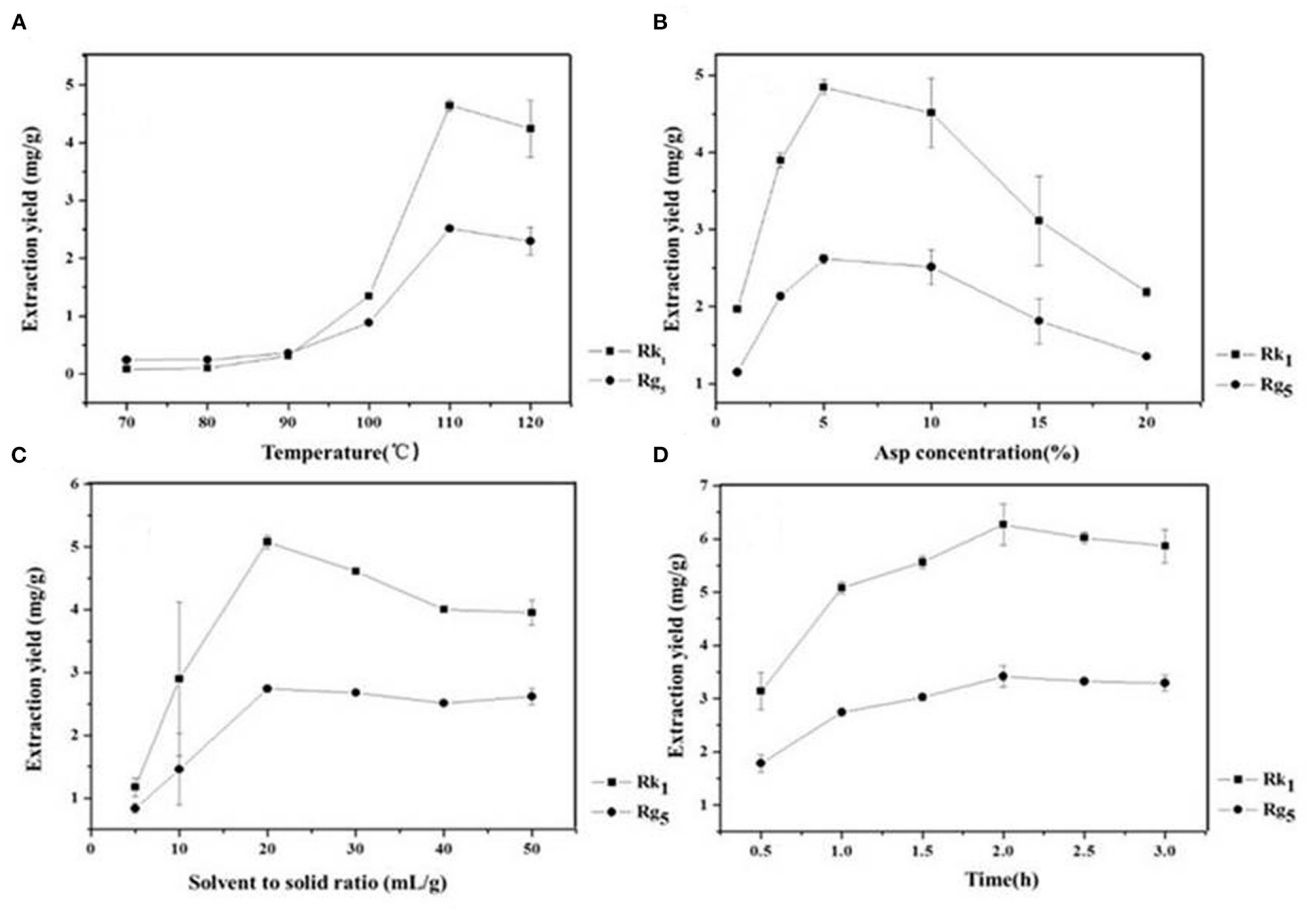
Effect of reaction **(A)** temperature **(B)** Asp concentration **(C)** liquid-solid ratio and **(D)** reaction time on the conversion content of rare ginsenosides Rk_1_ and Rg_5_ (*n* = 3).

Temperature is an important factor that affects the formation of rare ginsenosides. The results show that the conversion rates of the rare ginsenosides Rk_1_ and Rg_5_ increased gradually and tended to be stable (Figure 2A). At 110 °C, the conversion rates of Rk_1_ and Rg_5_ were the highest, 4.65 ± 0.10 mg/g and 2.52 ± 0.05 mg/g, respectively, which were significantly different from those of the first 4 groups (*P* < 0.05). Although the conversion rates at 110°C were higher than those achieved at 120°C, there was no significant difference between the two groups. Considering large-scale production, 110°C is easier to achieve with less energy consumption and a higher conversion rate. Therefore, 110°C was finally selected as the reaction temperature (Figure 2A). The concentration of the amino acid has a direct effect on the conversion rates of ginsenosides. The results showed that as the concentration of Asp increased (1–5%), the conversion rates of the rare ginsenosides Rk_1_ and Rg_5_ also increased, as shown in Figure 2B. With 5% Asp, the highest conversion rates of Rk_1_ and Rg_5_ were reached, 4.84 ± 0.09 mg/g and 2.62 ± 0.05 mg/g, respectively, and were significantly higher than those of the other groups (*P* < 0.05). When the concentration of the amino acid exceeded 5%, the conversion rates of the rare ginsenosides gradually decreased. Therefore, the optimum concentration of Asp was finally selected to be 5% (Figure 2B). The effect of the liquid-solid ratio on the conversion rates of the rare ginsenosides Rk_1_ and Rg_5_ is shown in Figure 2C. With an increase in the liquid-solid ratio, the conversion rates of Rk_1_ and Rg_5_ initially increased and then decreased. The conversion rates of Rk_1_ and Rg_5_ reached their highest values at 20 mL/g, 5.08 ± 0.11 mg/g and 2.74 ± 0.05 mg/g, respectively, and were significantly higher than those of other five groups (*P* < 0.05). The optimum liquid to solid ratio was finally selected to be 20 mL/g (Figure 2C). The effect of reaction time on the conversion rates of Rk_1_ and Rg_5_ is shown in Figure 2D. Rare ginsenosides, Rk_1_ and Rg_5_, gradually increased with increasing reaction time. The conversion rates of Rk_1_ and Rg_5_ reached their highest values at 2 h, 6.27 ± 0.39 mg/g and 3.42 ± 0.19 mg/g, respectively, and were significantly higher than those of the first three groups (*P* < 0.05). When the reaction time exceeded 2 h, the conversion rate of Rk_1_ and Rg_5_ did not continue to increase, and there was no significant difference between the conversion content obtained at 2 h (*P* < 0.05). This may be because as the reaction time increases, the substrate is continuously consumed, and the effective collision decreases after the active ingredient reaches the maximum value, so the content changes tend to remain the same. Considering the principle of large-scale production in the future, low energy consumption and high yield, 2 h was selected as the best reaction time (Figure 2D).

### Effect of Extraction Methods on the Conversion Rates

The choice of extraction method depends mainly on the advantages and disadvantages of each extraction technique and its impact on the extraction rate. The traditional extraction methods of ginsenosides mainly include decocting, dipping and reflow. In the Chinese pharmacopeia, ginsenosides are mainly treated by ultrasound, so we chose the above four methods RE, HE, ME, and USE for comparison. The results were are shown in Table 1. After adding the amino acids, HE produced the highest conversion rates of Rk_1_ and Rg_5_, which were 6.27 ± 0.39 mg/g and 3.42 ± 0.19 mg/g, respectively, RE was second best, and the conversion rates of USE were not ideal. The conversion of Rk_1_ and Rg_5_ by HE was twice as high as that by RE and four times as high as that by USE. This may be because temperature was an important factor in the conversion of rare ginsenosides by amino acids (23). Therefore, HE was more suitable for the conversion of Rk_1_ and Rg_5_, and HE was simple and easy to mass produce.

**Table 1:** Comparison of extraction methods.

**Method**	**Extraction yield (mg/g)**	
	**Ginsenoside Rk_**1**_**	**Ginsenoside Rg_**5**_**
RE	2.89 ± 1.22	1.45 ± 0.57
HE	6.27 ± 0.39	3.42 ± 0.19
ME	0.10 ± 0.02	0.24 ± 0.01
USE	1.35 ± 0.02	0.88 ± 0.01

### Orthogonal Test Results

**Figure 3:**
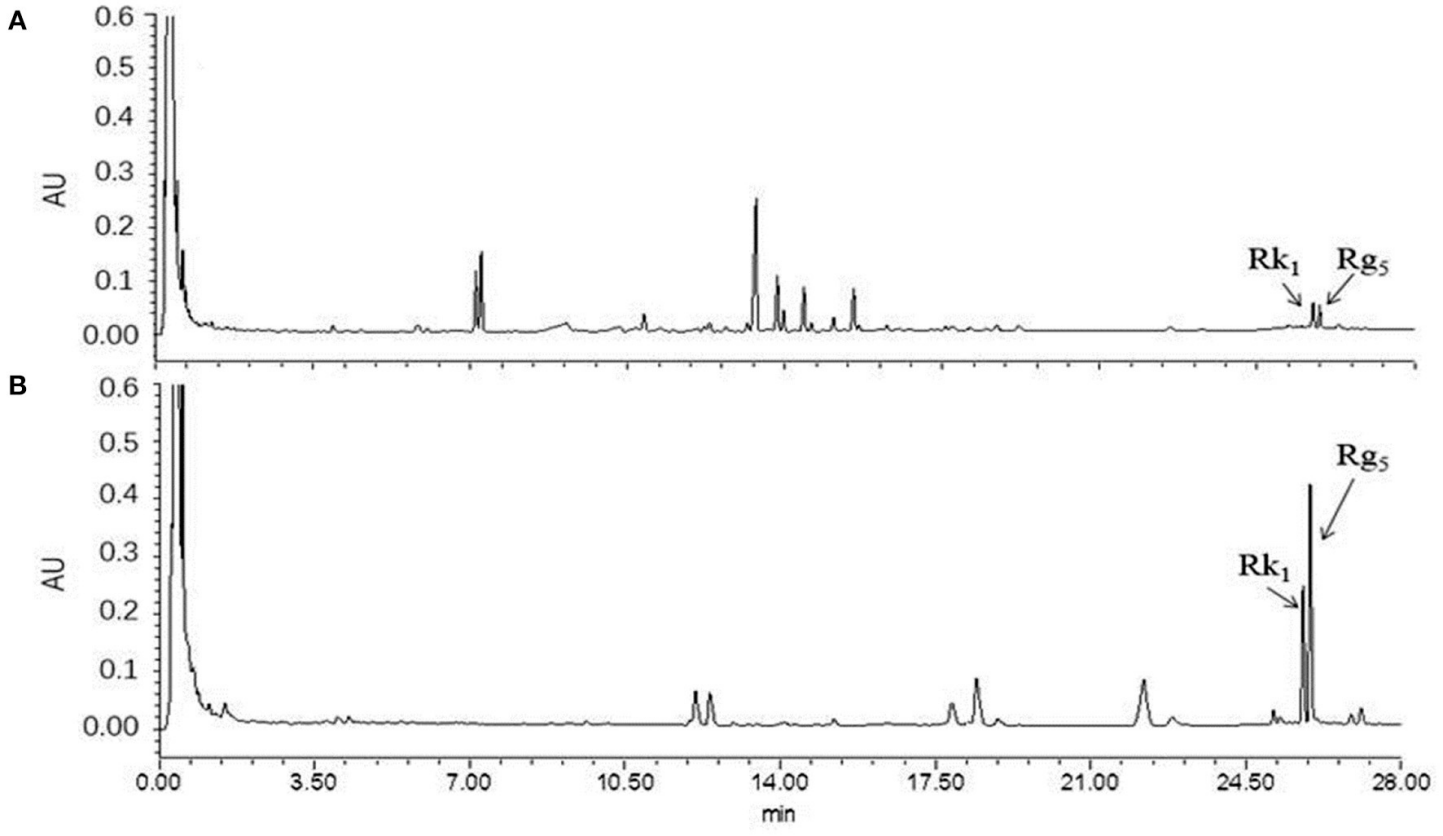
UPLC diagram before and after the reaction of American ginseng. **(A)** before reaction, **(B)** after reaction (*n* = 3).

Due to a number of factors needed for the conversion of rare ginsenosides, there are certain factors used to optimize the extraction process. Through the results of the previous single factor test, the final selection was based on the reaction temperature (°C), reaction time (h), amino acid concentration (%), and liquid-solid ratio (mL/g) as the investigation factors, and each factor was set at three levels. The L_9_ (3^4^) orthogonal test design table was carried out for nine tests. The orthogonal design and measurement results are shown in Tables 2, 3. Variance analysis results showed that the concentration of Asp and the reaction temperature had significant effects on the conversion rates of the rare ginsenosides Rk_1_ and Rg_5_ (*P* < 0.05) and the liquid-solid ratio and reaction time had no significant effect on the conversion rates. Range analysis was used to show that the effect of the four factors on Rk_1_ and Rg_5_. The results were Asp concentration>reaction temperature>solid-liquid ratio>time, and the *F value* result was consistent with the variance analysis. The optimal transformation conditions for amino acid hydrolysis of American ginseng to transform to the rare ginsenosides Rk_1_ and Rg_5_, are as follows: 5% Asp, 110°C reaction temperature, 30 mL/g solid-liquid ratio, and 2.5 h reaction time. The optimal conversion process was verified and the total content of Rk_1_ and Rg_5_ was 10.25 ± 0.32 mg/g. The UPLC comparison chart of American ginseng before and after the reaction is shown in Figure 3.

**Table 2:** Orthogonal test design and results.

**Desgin**	**Factor**				**Extraction**	
**ID number**					**yield (mg/g)**	
	**(A)**	**(B) Asp**	**(C) Solvent**	**(D)**	**Rk**_**1**_	**Rg**_**5**_
	**Temperature**	**concentration**	**to solid**	**Time**
	**(** **°** **C)**	**(%)**	**ratio (mL/g)**	**(h)**
1	A_1_ = 100	B_1_ = 3	C_1_ = 10	D_1_ = 2	2.37	1.65
2	A_1_ = 100	B_2_ = 5	C_2_ = 20	D_2_ = 2.5	3.94	2.88
3	A_1_ = 100	B_3_ = 10	C_3_ = 30	D_3_ = 3	3.22	2.49
4	A_2_ = 110	B_1_ = 3	C_2_ = 20	D_3_ = 3	4.35	2.85
5	A_2_ = 110	B_2_ = 5	C_3_ = 30	D_1_ = 2	5.84	4.08
6	A_2_ = 110	B_3_ = 10	C_1_ = 10	D_2_ = 2.5	4.83	3.07
7	A_3_ = 120	B_1_ = 3	C_3_ = 30	D_2_ = 2.5	4.07	2.79
8	A_3_ = 120	B_2_ = 5	C_1_ = 10	D_3_ = 3	5.14	3.45
9	A_3_ = 120	B_3_ = 10	C_2_ = 20	D_1_ = 2	2.85	2.37
K_1_	5.52	6.04	6.84	6.39		
K_2_	8.34	8.44	6.42	7.19		
K_3_	6.89	6.27	7.49	7.18		
R	2.82	2.40	1.07	0.80		

**Table 3:** Analysis of variance of orthogonal test.

**Factors**	**SS**	**df**	**MS**	**F**	**P**	**Significant**
A	11.960	2	5.9800	7.928	0.0406	^*^
B	10.509	2	5.2545	6.967	0.0497	^*^
C	1.747	2	0.8735	1.158	0.401	
D	1.270	2	0.6350	0.842	0.495	
Erro	3.02	4				
Total	25.486	8				

*F_0.05_ (2,4) = 6.940*

*SS, Sum of square; df, Degree of freedom; MS, Mean of square; F, F-value*.

* *P < 0.05 (F >F _0.05_ (2,4), (A) Temperature (B) Asp Concentration (C) Solvent to solid ratio (D) Time*.

### Confirmation of Transformation Pathway

**Figure 4:**
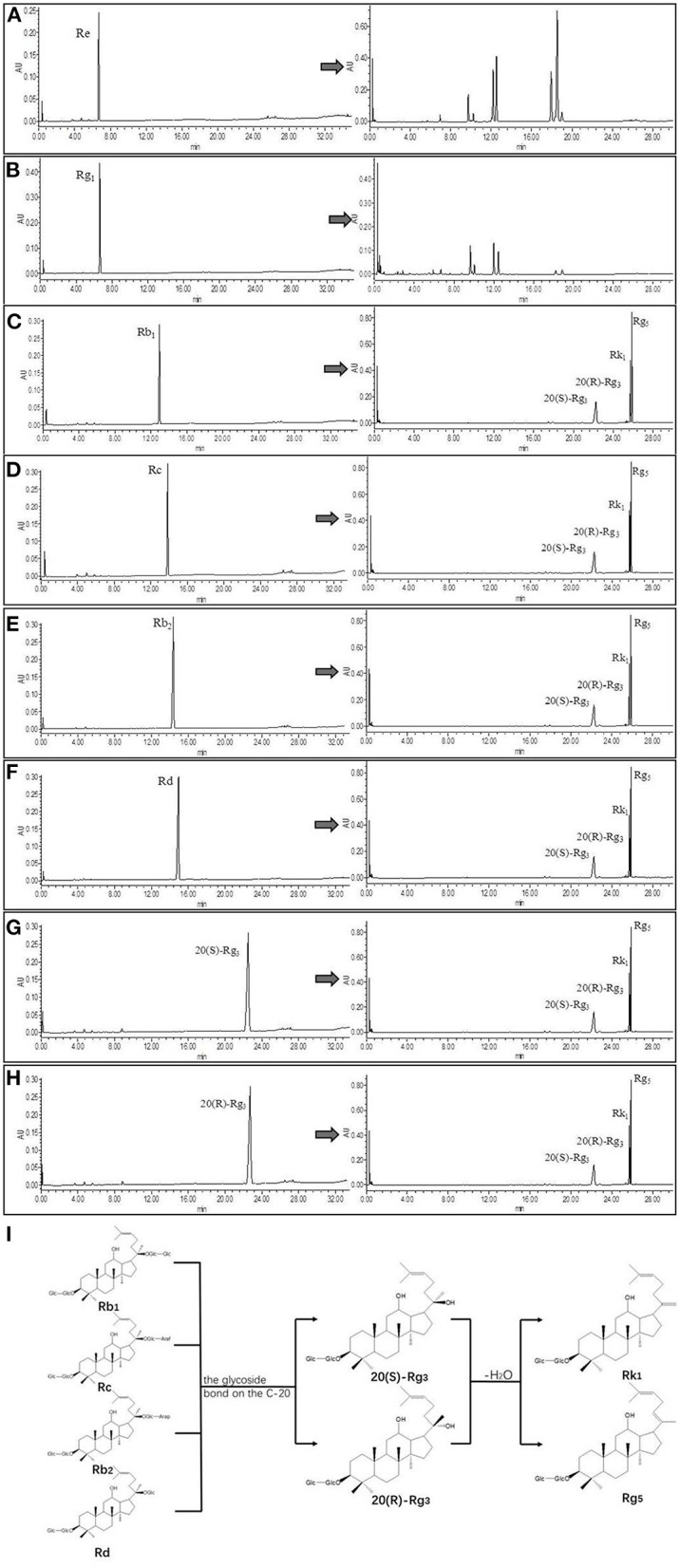
UPLC comparison chart before and after monomeric saponin conversion (*n* = 3). **(A–H)** is a comparison of UPLC before and after transformation of ginsenoside Re, ginsenoside Rg_1_, ginsenoside Rb_1_, ginsenoside Rc, ginsenoside Rb_2_, ginsenoside Rd, ginsenoside 20(S)-Rg_3_, and ginsenoside 20(R)-Rg_3_. **(I)** Transformation pathway of ginsenosides Rk_1_ and Rg_5_ in protopanaxadiol saponins.

Referring to the experimental results of Liu Z (16, 24), we selected eight pure ginsenosides to confirm the transformation pathway of the rare ginsenosides Rk_1_ and Rg_5_. The UPLC comparison before and after the conversion of pure ginsenosides is shown in Figure 4. The PPD types, Rc, Rd, Rb_1_, and Rb_2_ were completely converted into the rare ginsenosides 20(S)-Rg_3_, 20(R)-Rg_3_, Rk_1_, and Rg_5_ under the optimal conditions of this experiment. Under these conditions, ginsenosides 20(S)-Rg_3_ and 20(R)-Rg_3_ were partially converted to Rk_1_ and Rg_5_; the PPT types Rg_1_ and Re did not generate Rk_1_ and Rg_5_. This is consistent with the possible reaction pathway of ginsenosides as explained in the ginseng pyrolysis study (20). The reaction pathway is shown in Figure 4. The PPD types cleave at the C-20 position to form the intermediate products 20(S)-Rg_3_ and 20(R)-Rg_3_, and further dehydration and hydrolysis occur to transform them to Rk_1_ and Rg_5_. In this paper, the addition of Asp increased the yield of rare ginsenosides. This may be due to the fact Asp is an acidic amino acid and H^+^ promotes the hydrolysis reaction (25). Moreover, the content of PPD types is high in American ginseng, which provides the necessary conditions for the conversion of rare ginsenosides.

### Effect of AGS Synergistic CTX on S180 Tumor-Bearing Mice on Tumor Inhibition and Immune Indices

**Figure 5:**
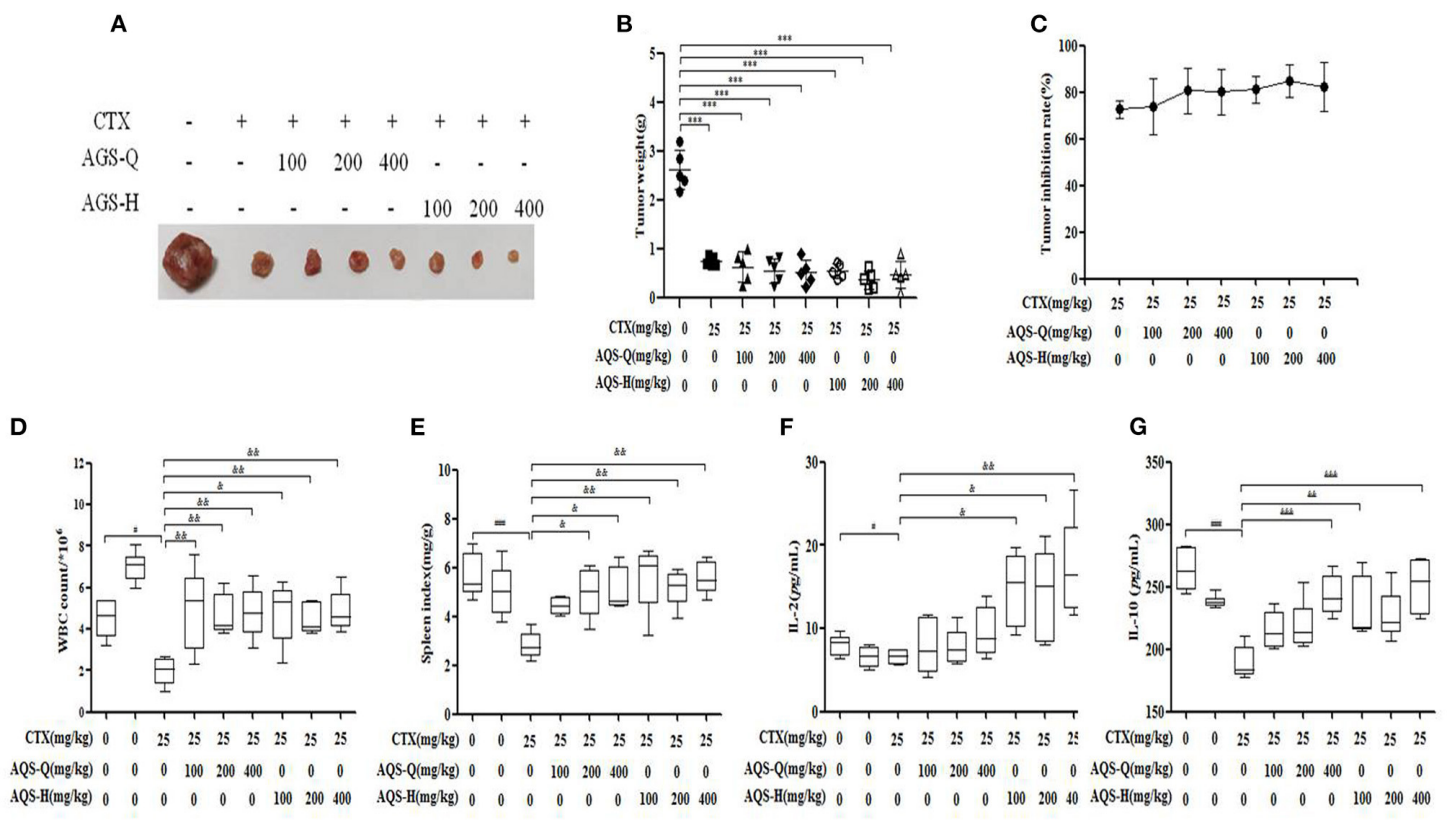
Effects of AGS-Q or AGS-H synergistic CTX on tumor inhibition and immune indexes (*n* = 8). **(A)** Tumor image. **(B)** Tumor weight. **(C)** Tumor inhibition rate. **(D)** The white blood cells count. **(E)** Spleen index. **(F)** The content of IL-2. **(G)** The content of IL-2. Values are presented as mean ± SD. vs. normal group, ^*##*^*P* < 0.01; vs. model group, ***P < 0.001; vs. CTX group, ^&^*P* < 0.05, ^&&^*P* < 0.01, ^&*&&*^*P* < 0.001.

CTX is a broad-spectrum anticancer drug that has a good inhibitory effect on malignant tumors, but has obvious toxic side effects. It can cause loss of appetite, nausea, weight loss and adverse reactions such as decreased white blood cells (23). IL-2 and IL-10 are a type of cell growth factor in the immune system that can regulate the cell activity of white blood cells in the immune system, promote the proliferation of Th0 cells and CTLs, and also participate in the antibody response, haematopoiesis and tumor surveillance (26). Compared with the model group, mice treated with CTX, American ginseng total saponins (AGS-Q) or American ginseng total saponins after transformation (AGS-H) synergistic CTX can significantly reduced tumor weights, as shown in Figure 5B (*P* < 0.001). Compared with the normal group, CTX significantly reduced the white blood cell count, spleen index and IL-2 and IL-10 content (*P* < 0.05, *P* < 0.001), which showed obvious immunosuppressive side effects. AGS-Q synergistic CTX enhanced the spleen index, white blood cell count and IL-10 content compared with the CTX group (*P* < 0.05, *P* < 0.001). AGS-H synergistic CTX enhanced the spleen index, white blood cell count, and IL-2 and IL-10 contents compared with the CTX group (*P* < 0.05, *P* < 0.001). The AGS-H synergistic CTX group improved the immune indices better than the AGS-Q group.

### Effect of AGS Synergistic CTX on Splenic T-Lymphocyte Subpopulations

**Figure 6:**
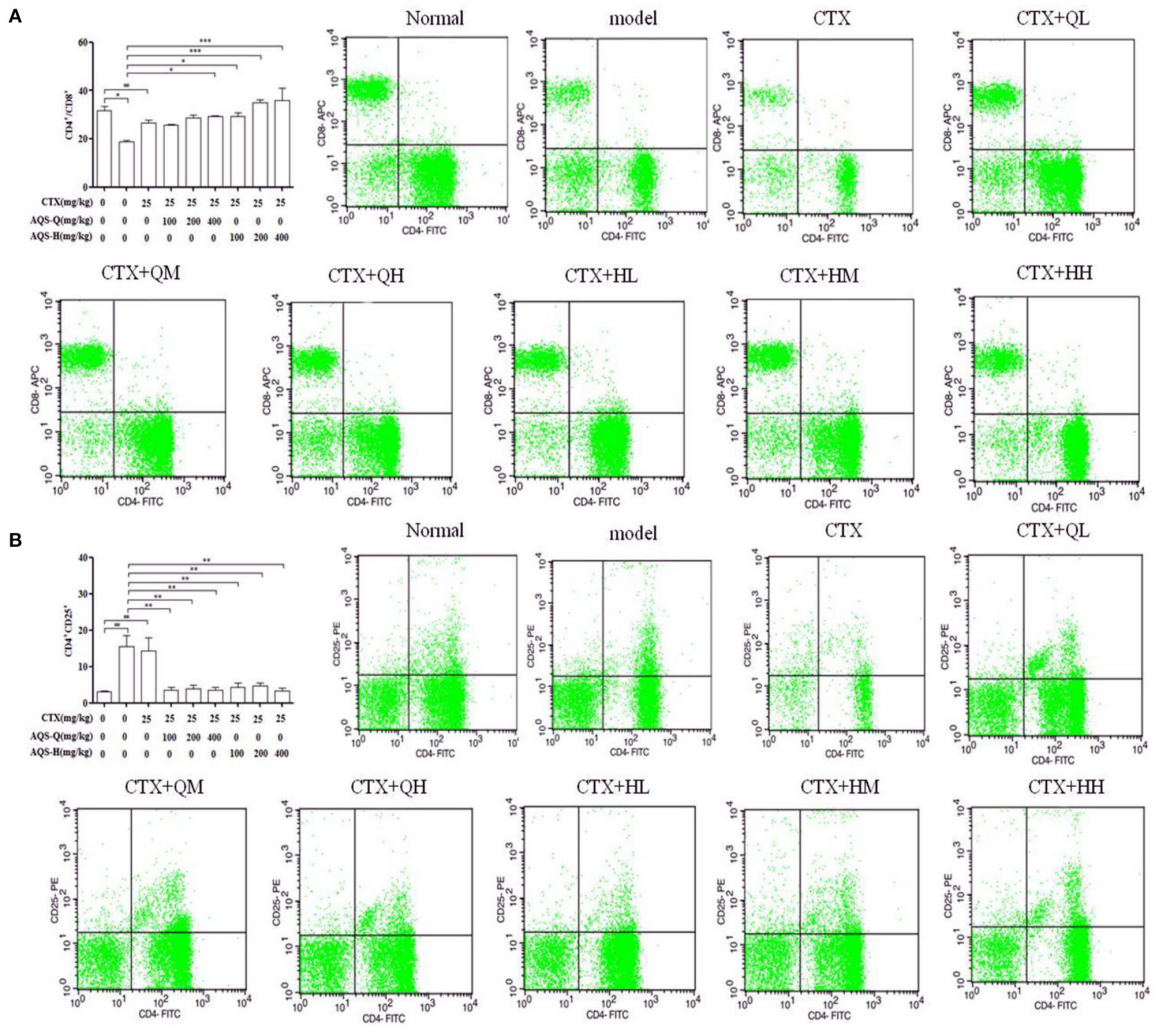
Flow scatter plot of T lymphocyte subsets (*n* = 8). Percentage of T lymphocyte subsets and **(A)** CD4^+^/CD8^+^, **(B)** CD4^+^CD25^+^ T lymphocyte ratio. Values are presented as mean ± SD. vs. normal group, ^##^*P* < 0.01, ^###^*P* < 0.001; vs. model group, *P < 0.05, ***P < 0.001.

Splenocytes consist of various immune cells, including T or B lymphocytes, macrophages, and dendritic cells. T cell subpopulations have a great importance in T cell homeostasis and immune regulation, and the T lymphocyte phenotype is mainly divided into CD3^+^, CD4^+^ and CD8^+^ T cells (27). Compared with the normal group in Figure 6, CTX significantly decreased the CD4^+^/CD8^+^ ratio, and enhanced the CD4^+^CD25^+^ content (*P* < 0.001). Compared with the model group in Figure 6, AGS-Q synergistic CTX can significantly restored the ratio of CD4^+^/CD8^+^ cells and significantly inhibited the level of CD4^+^CD25^+^ (*P* < 0.001). AGS-H synergistic CTX can significantly restored the ratio of CD4^+^/CD8^+^ cells and significantly inhibited the level of CD4^+^CD25^+^ (*P* < 0.001). The results indicated that the AGS can restore the damaged splenic T lymphocyte subsets.

### Effect of AGS Synergistic With CTX on Tumor Apoptosis in S180 Tumor-Bearing Mice

**Figure 7:**
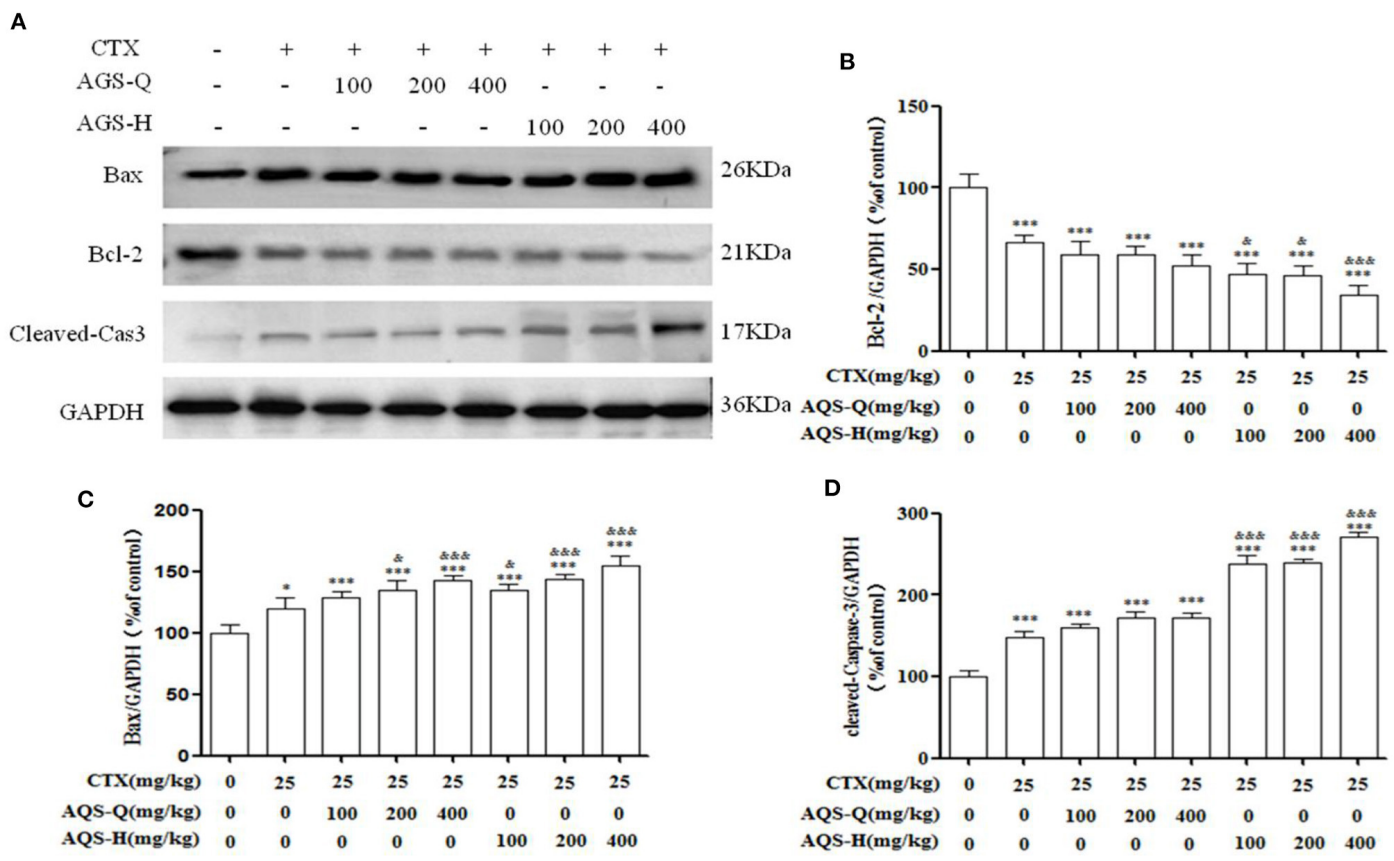
Effects of AGS synergistic CTX on Bcl-2, Bax and cleaved-Caspase-3 in S180 tumor-bearing mice (*n* = 8). **(A)** The protein expression levels of Bcl-2, Bax and cleaved-Caspase-3 were detected by Western blotting. **(B)** The relative Bcl-2 expression level. **(C)** The relative Bax expression level. **(D)** The relative cleaved-Caspase-3 expression level. Values are presented as mean ± SD. vs. model group, *P < 0.05, **P < 0.01, ***P < 0.001; vs. CTX group, ^&^*P* < 0.05, ^&&^*P* < 0.01, ^&*&&*^*P* < 0.001.

In order to explore the effect of AGS synergistic with CTX on the tumor apoptosis. We detected the effects of AGS-Q and AGS-H on Bax, Bcl-2 and cleaved-Caspase-3 proteins in tumor tissue (Figure 7). Compared with the model group, CTX significantly upregulated the expression of Bax and cleaved-Caspase-3, and inhibited the expression of antiapoptotic protein Bcl-2 (*P* < 0.05, *P* < 0.001). AGS-Q or AGS-H synergistic with CTX significantly upregulated the expression of Bax and cleaved-Caspase-3, and inhibited the expression of antiapoptotic protein Bcl-2 in a dose-dependent manner (*P* < 0.001). In comparison with CTX, AGS-Q synergistic with CTX increased the expression of Bax. AGS-H synergistic with CTX significantly inhibited the expression of Bcl-2 and promoted Bax and cleaved-Caspase-3 in tumor tissues (*P* < 0.05, *P* < 0.001). The AGS-H synergistic CTX group had a higher level of promoting tumor apoptosis protein expression than the AGS-Q synergistic CTX group.

### Effect of AGS-H Synergistic CTX/2 on Tumor Inhibition and Immune Indices

**Figure 8:**
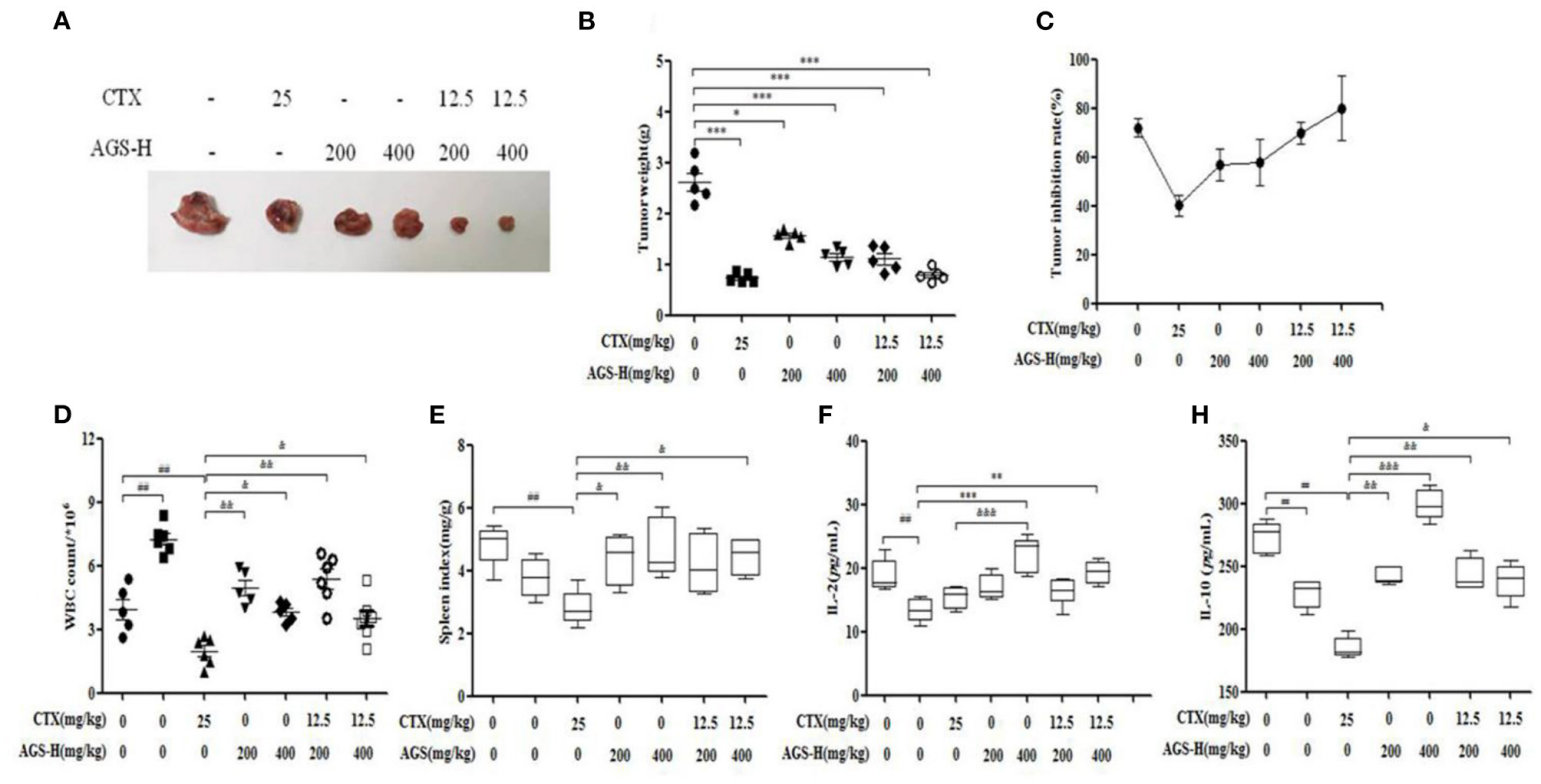
Effects of CTX/2 + AGS-H on tumor inhibition and immune indexes (*n* = 8). **(A)** Tumor image. **(B)** Tumor weight. **(C)** Tumor inhibition rate. **(D)** The white blood cells count. **(E)** Spleen index. **(F)** The content of IL-2. **(G)** The content of IL-10. Values are presented as mean ± SD. vs. normal group, ^*##*^*P* < 0.01; vs. model group, *P < 0.05, **P < 0.01, ***P < 0.001; vs. CTX group, ^&^*P* < 0.05 ^&&^*P* < 0.01, ^&*&&*^*P* < 0.001.

The tumor weight in the model group was more than 1 g, indicating that S180 tumor-bearing mice were successfully inoculated. Compared with the model group, the CTX, AGS-L and AGS-H groups had significantly reduced tumor weight and inhibited tumor growth (*P* < 0.05, *P* < 0.001). CTX/2+AGS-HL or AGS-HH significantly reduced tumor weight (Figures 8A,B). The spleen and white blood cells are an important part of the immune system and protect the body from infectious diseases and pathogens. Compared with the normal group, the number of white blood cells significantly increased, and the levels of the immune factors IL-2 and IL-10 were significantly decreased in the model group (*P* < 0.05, *P* < 0.01, *P* < 0.001). CTX significantly reduced white blood cell counts, spleen index and IL-10 levels in mice (*P* < 0.01). Compared with the CTX group, AGS-HL, AGS-HH and CTX/2+AGS-HL or HH significantly increased the white blood cell count and serum IL-10 content (*P* < 0.05, *P* < 0.01, *P* < 0.001). AGS-HL, AGS-HH and CTX/2+AGS-HH increased the spleen index (*P* < 0.05, *P* < 0.01). AGS-HH significantly increased IL-2 levels (*P* < 0.001). These results suggest that AGS-H can improve the development of immune organs in tumor-bearing mice, upregulate the number of white blood cells to enhance cellular immunity, and regulate the effects of the immune factors IL-2 and IL-10.

### Effects of AGS-H Synergistic CTX/2 on Lymphocyte Subsets of S180 Tumor-Bearing Mice

**Figure 9:**
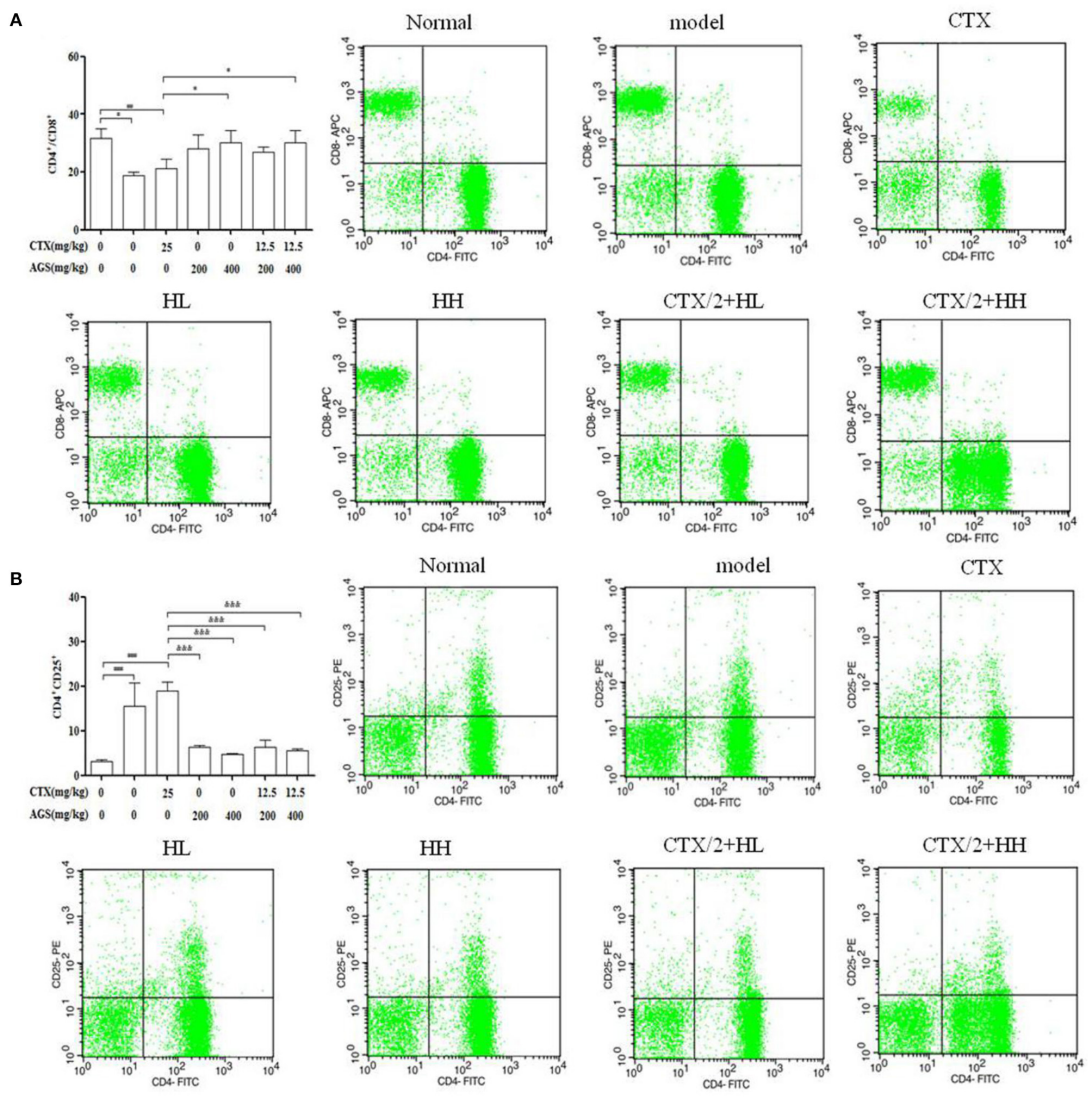
Effects of AGS-H synergistic CTX/2 on lymphocyte subsets in S180 tumor-bearing mice (*n* = 8). **(A)** CD4^+^/CD8^+^. **(B)** CD4^+^CD25^+^. Values are presented as mean ± SD. vs. normal group, ^#^*P* < 0.05, ^*###*^*P* < 0.001; vs. model group, *P < 0.05, **P < 0.01, ***P < 0.001; vs. CTX group, ^&^*P* < 0.05, ^&&^*P* < 0.01, ^&*&&*^*P* <‘0.001.

In Figure 9, compared with the normal group, we observed that the CD4^+^/CD8^+^ T cell ratio was significantly decreased, and the content of CD4^+^CD25^+^ T cells was significantly increased in the model and CTX groups (*P* < 0.05, *P* < 0.001). Compared with the CTX group, AGS-HH and AGS-HH synergistic CTX/2 treatment slightly enhanced CD4^+^/CD8^+^ (*P* < 0.05). Treatment with AGS-HL and AGS-HH caused a significant decrease of CD4^+^CD25^+^ (*P* < 0.001). Treatment with AGS-HL or AGS-HH synergistic CTX/2 caused a significant decrease in CD4^+^CD25^+^ (*P* < 0.001). These results indicated that the AGS-H synergistic with CTX/2 administration group could restore the damaged T lymphocyte subsets of the spleen.

### Effects of AGS-H Synergistic CTX/2 on Apoptotic Proteins in S180 Tumor-Bearing Mice

**Figure 10:**
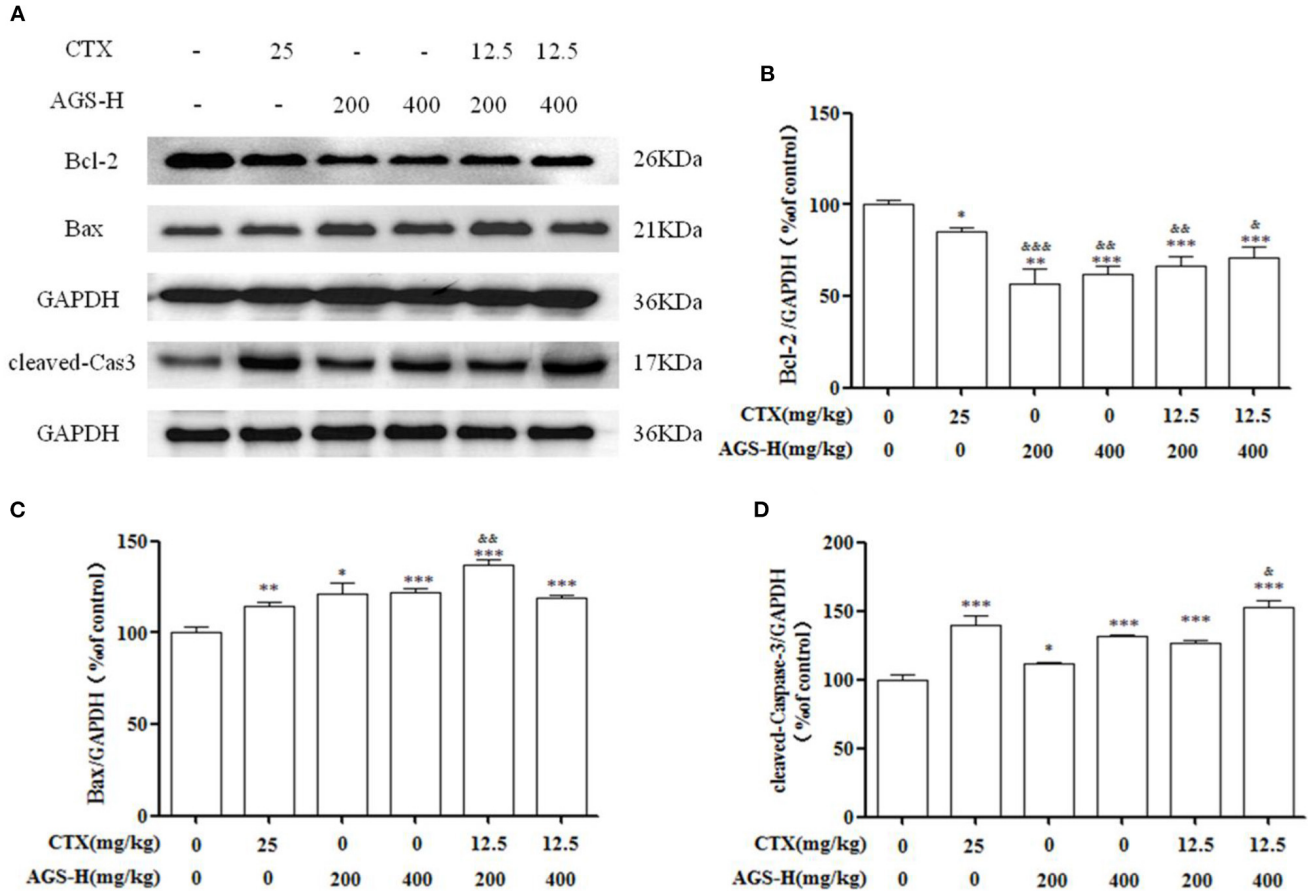
Effects of AGS-H synergistic CTX/2 on Bcl-2, Bax and cleaved Caspase-3 in S180 tumor-bearing mice (*n* = 8). **(A)** The protein expression levels of Bcl-2, Bax and cleaved-Caspase-3 were detected by Western blotting. **(B)** The relative Bcl-2 expression level. **(C)** The relative Bax expression level. **(D)** The relative cleaved-Caspase-3 expression level. Values are presented as mean ±SD. vs. model group, *P < 0.05, ***P < 0.001; vs. CTX group, ^&^*P* < 0.05, ^&&^*P* < 0.01, ^&*&&*^*P* < 0.001.

As shown in Figure 10, compared with the S180 tumor-bearing mice model group, the expression of the antiapoptotic factor Bcl-2 was decreased in the AGS-H and AGS-H synergistic with CTX/2 groups (*P* < 0.05, *P* < 0.001), which significantly increased the expression of the apoptosis factors Bax and cleaved-Caspase-3 (*P* < 0.05, *P* < 0.001) in a dose-dependent manner. Compared with the CTX group, AGS-H and AGS-H synergistic CTX/2 treatment significantly inhibited the level of Bcl-2 in tumor tissues. AGS-HL synergistic CTX/2 significantly promoted the expression of Bax (*P* < 0.01). AGS-HH synergistic with CTX/2 increased the content of cleaved-Caspase-3 (*P* < 0.05). The results showed that AGS-H synergistic CTX/2 administration induced apoptosis in S180 tumor tissue.

## Discussion

Ginsenosides are the main active components in *Panax* ginseng and *Panax quinquefolium* L. With different processing methods, the contents and types of ginsenosides in ginseng also changed, and new ginsenosides were generated. Studies have shown that cooking ginseng at high temperature can change the species of ginsenosides and transform them into less polar ginsenosides. In acidic environments, ginsenosides are usually transformed by deglycosylation (24, 28). Therefore, temperature and pH value are the key factors for the transformation of ginsenosides. Liu discussed that aspartic acid could effectively degrade ginsenosides into rare ginsenosides, and the concentration of ginsenoside Rg5 increased with temperature, which also proved that ginsenosides could be transformed into rare ginsenosides by heating and adding aspartic acid (16).

In our study, the effects of the types of amino acids and extraction methods on the conversion of common ginsenosides into rare ginsenosides Rk_1_ and Rg_5_ were investigated, and the reaction temperature (°C), amino acid concentration (%), reaction time (h) and liquid-solid ratio were further discussed using orthogonal experiments (mL/g). The effect of its conversion rate, the reaction pathway and mechanism are preliminarily discussed using monomeric ginsenoside as the substrate. In variance analysis, we found that temperature and the amount of amino acids had the most important influence on the transformation of rare ginsenosides. The results show that Asp is the best catalyst, and thermal extraction has the best effect. The addition of aspartic acid makes the solution weakly acidic, which is also the reason for the transformation of a large number of rare ginsenosides. Under the optimal conversion conditions (110°C, 5% Asp, 2.5 h reaction time, 30 mL/g), the highest conversion rates of rare ginsenosides Rk_1_ and Rg_5_ were 6.58 ± 0.11 mg/g and 3.74 ± 0.05 mg/g, respectively. In the reaction pathway, the saponins of the ginsengdiol group mainly participate in the transformation process, and the saponins of the ginsengtriol group basically do not participate in the transformation process. Compared with existing transformation methods such as enzymatic acid hydrolysis, the food-grade amino acid transformation method is simpler, more feasible, safe and effective, and provides the possibility for the large-scale production and preparation of rare ginsenosides Rk_1_ and Rg_5_. Therefore, the use of Asp will improve American ginseng transformation of rare ginsenosides and is of great significance.

Ginsenosides have a good immunomodulatory and anticancer effects. CTX is a broad-spectrum anticancer drug that has a good inhibitory effect on malignant tumors, but has obvious toxicity and side effects, including immunosuppression, and adverse reactions such as loss of appetite, nausea, weight loss and leukopenia (29). In this study, combined with the deficiency of CTX in tumor treatment and the advantages of AGS-Q and AGS-H in antitumour and immune regulation, CTX combined with AGS-Q and AGS-H was used to treat S180 tumor-bearing mice, giving full play to the advantages of chemotherapy and Chinese herbal medicine. We conducted experiments on the effect of AGS-Q or AGS-H synergistic CTX on S180 tumor-bearing mice. The results showed that AGS-Q or AGS-H synergistic CTX in each dose group can significantly inhibit tumor-bearing growth, and upregulate white blood cells and spleen index in comparison with CTX. Additionally, AGS-H synergistic CTX/2 treatment of S180 tumor-bearing mice was found to inhibit tumor growth and restore the immune organ index and white blood cell number.

The imbalance of T lymphocyte subsets may lead to immune dysfunction, leading to a series of immune responses and immunopathological changes (30). During tumor immunity, the function of helper CD4^+^ T cells is weakened, while the immunosuppressive function of regulatory T cells is enhanced (31). CD4^+^ is a marker on the surface of helper T cells (Th cells), which play an important role in enhancing humoral and cellular immunity. CD8^+^ T cells are a marker of inhibitory T cells (Ts cells) that specifically kill infected and dysfunctional cells (27). The increase in the CD4^+^/CD8^+^ ratio indicates that Th cells are higher than inhibitory T cells, indicating that the immune capacity of the body is improved. Treg cells, as the main immunosuppressive cells in mouse and human tumors, are T cell subsets with phenotypic characteristics of CD4^+^CD25^+^Foxp3^+^, which play an important role in regulating the tumor microenvironment and promoting tumor immune avoidance (32). Cytokines are small active molecules produced by immune cells. Cytokines have strong immunomodulatory effects and play an important role in tumor immunity (33). The cytokine IL-2 is a growth factor of T cells and plays an important role in the activation and proliferation of T cells, the activation of B cells and macrophages, and the secretion of IL-2 by T cells. IL-2 mainly plays an immune promotion role *in vivo* and can promote the antitumour immunity of immune cells (34, 35). Interleukin-10 (IL-10) is a cytokine widely expressed in T cells, B lymphocytes, mononuclear macrophages and keratin cells, and has both promoting and inhibiting effects on tumors (36). In this study, AGS-Q or AGS-H synergistic with CTX and AGS-H synergistic with CTX/2 combined activated the immune system of S180 mice, increased the ratio of CD4^+^/CD8^+^ and reduced the number of CD4^+^CD25^+^ cells. Activated CD4^+^ T cells secrete IL-2. IL-10 release catalyses an immune response that inhibits tumor growth. AGS-H synergistic CTX showed better tumor suppression than AGS-Q synergistic with CTX and reduced the immunosuppression induced by CTX. AGS-H synergistic with CTX/2 treatment of S180 tumor-bearing mice has an equivalent tumor suppressive effect to CTX and meanwhile can reduce the immunosuppressive effect of CTX.

The occurrence of tumor diseases is a sign of the unregulation of cell apoptosis, which makes some abnormal or senescent cells immune escape and enter a state of immortal proliferation. Changes in cell apoptosis are related to the occurrence and development of tumors. Members of the Bcl-2 family are important regulators of the mitochondrial apoptosis pathway (37). According to their different functions, they can be divided into the antiapoptotic protein family and the proapoptotic protein family. Bcl-2 is the main representative member of the antiapoptotic protein family, and Bax is one of the main representative proteins of the proapoptotic protein family (38, 39). Studies have shown that the Bcl-2 protein is highly expressed in many tumor cells, is the main antiapoptotic protein for sudden mitochondrial apoptosis, and plays an important role in the regulation of tumor cell apoptosis (40). In this study, compared with the CTX alone group, AGS synergistic with CTX or CTX/2 significantly upregulated the expression of Bax and cleaved-Caspase-3 and inhibited the expression of the antiapoptotic protein Bcl-2.

## Conclusion

This study provides a safe, green and effective transformation method for the enrichment of rare ginsenosides and provides a new idea for the development and utilization of American ginseng. AGS can not only play an antitumour role, but also reduce the side effects of immunosuppression caused by CTX, improve the immune activity of S180 tumor-bearing mice and promote the apoptosis of tumor cells. AGS-H is more effective in enhancing anticancer activity and immunity may be closely related to the increase in rare ginsenosides Rk_1_ and Rg_5_.

## Data Availability Statement

The original contributions presented in the study are included in the article/Supplementary Material, further inquiries can be directed to the corresponding authors.

## Ethics Statement

All experiments were executed strictly according with the Principle of Laboratory Animal Care and the guidelines prescribed by the Animal Research Committee of the Institute of Special Animals and Plants Sciences, Chinese Academy of Agricultural Sciences (Permit No.: ECLA-ISAP-18079).

## Author Contributions

Z-mL and Z-jS performed the implementation of animal experiments and wrote the manuscript. DQ, J-YS, and X-hH analyzed the data. MH and J-bC guided the transformation conditions. Y-sL supervised HPLC conditions and results. Y-sS and S-sL participated in the overall design guidance and manuscript review. All authors contributed to the article and approved the submitted version.

## Funding

This work was supported by National Natural Science Foundation of China (Grant No. 31200261), Jilin province science and technology development project (Grant No. 20200708070YY), and Central Public-interest Scientific Institution Basal Research Fund (Grant Nos. 1610342019008, 1610342020005, and CAAS-ASTIP-ISAPS-2021-010).

## Conflict of Interest

The authors declare that the research was conducted in the absence of any commercial or financial relationships that could be construed as a potential conflict of interest. The handling editor declared a shared affiliation with the authors at time of review.

## Publisher's Note

All claims expressed in this article are solely those of the authors and do not necessarily represent those of their affiliated organizations, or those of the publisher, the editors and the reviewers. Any product that may be evaluated in this article, or claim that may be made by its manufacturer, is not guaranteed or endorsed by the publisher.
