# Supplementary figures and images for: Transformation Mechanism of Rare Ginsenosides in American Ginseng by Different Processing Methods and Antitumour Effects
Source: Front Nutr. 2022 Apr 4;9:833859. doi: 10.3389/fnut.2022.833859 (PMC9014012; doi:10.3389/fnut.2022.833859)

FIGURE7

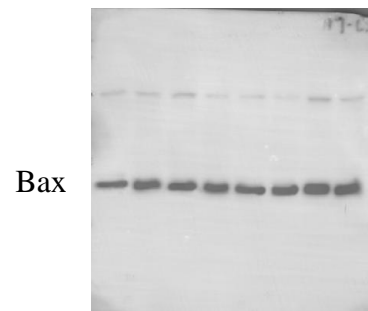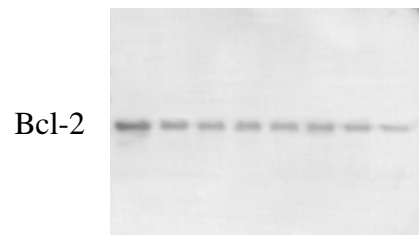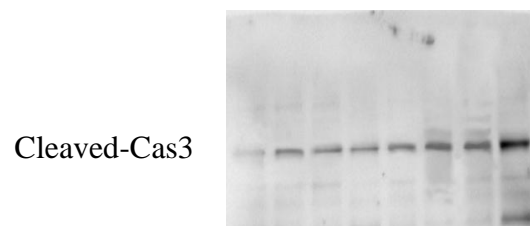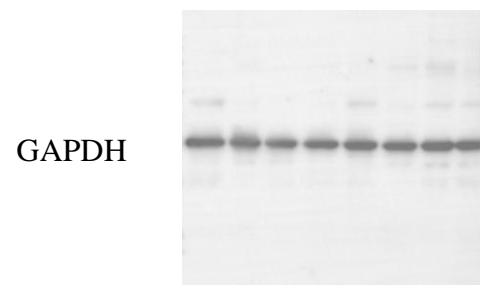

FIGURE10

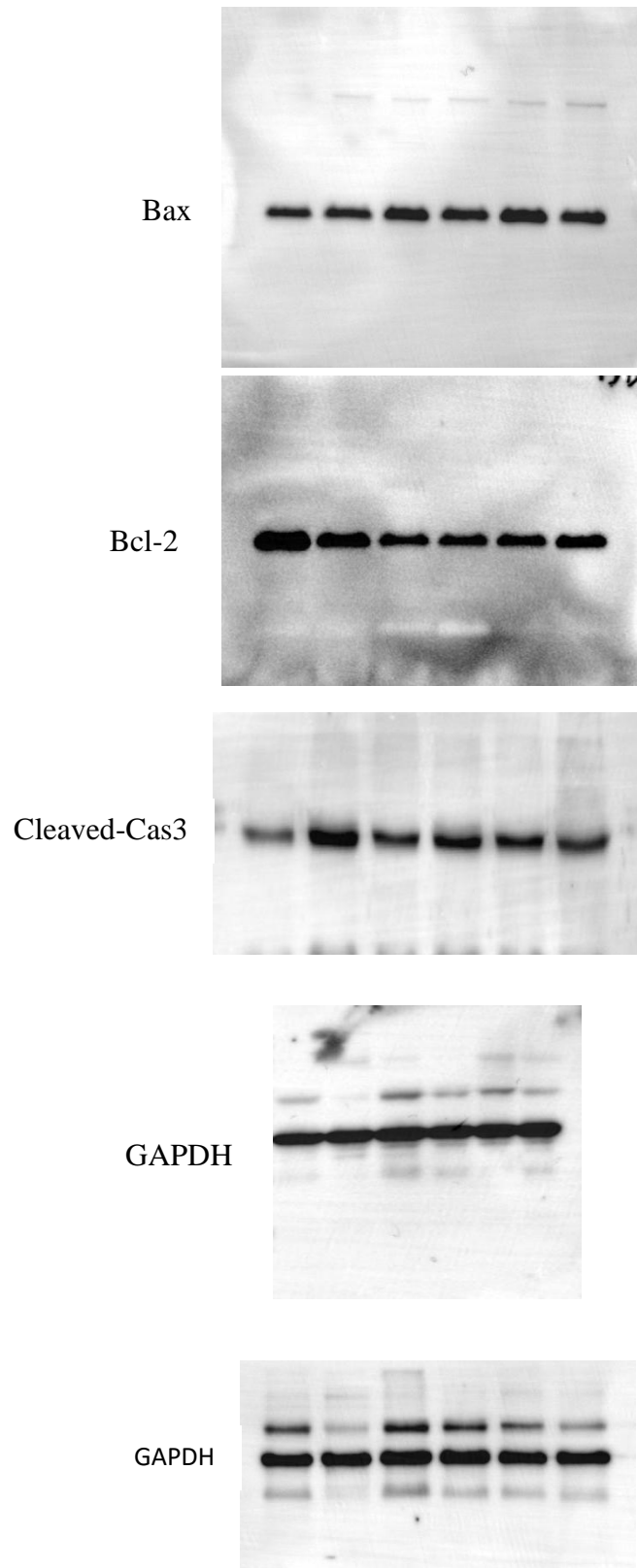

Supplement: Supplementary file 2 [file Data_Sheet_1.pdf]
